# Engineered Highly Porous Polyvinyl Alcohol Hydrogels with Poly(3-hydroxybutyrate-co-3-hydroxyvalerate) and Graphene Nanosheets for Musculoskeletal Tissue Engineering: Morphology, Water Sorption, Thermal, Mechanical, Electrical Properties, and Biocompatibility

**DOI:** 10.3390/ma16083114

**Published:** 2023-04-15

**Authors:** José Luis Aparicio-Collado, Qiqi Zheng, José Molina-Mateo, Constantino Torregrosa Cabanilles, Ana Vidaurre, Ángel Serrano-Aroca, Roser Sabater i Serra

**Affiliations:** 1 Centre for Biomaterials and Tissue Engineering, Universitat Politècnica de València, 46022 València, Spain; joapcol@upvnet.upv.es (J.L.A.-C.); jmmateo@fis.upv.es (J.M.-M.); ctorregr@fis.upv.es (C.T.C.); vidaurre@fis.upv.es (A.V.); 2 Biomedical Research Networking Centre in Bioengineering, Biomaterials and Nanomedicine (CIBER-BBN), 46022 València, Spain; 3 Biomaterials and Bioengineering Lab, Centro de Investigación Traslacional San Alberto Magno, Universidad Católica de Valencia San Vicente Mártir, 46001 València, Spain; 4 Department of Electrical Engineering, Universitat Politècnica de València, 46022 València, Spain

**Keywords:** carbon-based nanocomposite, conductive cell substrate, semi-IPN hydrogel, graphene nanosheets, poly(3-hydroxybutyrate-co-3-hydroxyvalerate), polyvinyl alcohol

## Abstract

Electroactive composite materials are very promising for musculoskeletal tissue engineering because they can be applied in combination with electrostimulation. In this context, novel graphene-based poly(3-hydroxybutyrate-co-3-hydroxyvalerate)/polyvinyl alcohol (PHBV/PVA) semi-interpenetrated networks (semi-IPN) hydrogels were engineered with low amounts of graphene (G) nanosheets dispersed within the polymer matrix to endow them with electroactive properties. The nanohybrid hydrogels, obtained by applying a hybrid solvent casting–freeze-drying method, show an interconnected porous structure and a high water-absorption capacity (swelling degree > 1200%). The thermal characterization indicates that the structure presents microphase separation, with PHBV microdomains located between the PVA network. The PHBV chains located in the microdomains are able to crystallize; even more after the addition of G nanosheets, which act as a nucleating agent. Thermogravimetric analysis indicates that the degradation profile of the semi-IPN is located between those of the neat components, with an improved thermal stability at high temperatures (>450 °C) after the addition of G nanosheets. The mechanical (complex modulus) and electrical properties (surface conductivity) significantly increase in the nanohybrid hydrogels with 0.2% of G nanosheets. Nevertheless, when the amount of G nanoparticles increases fourfold (0.8%), the mechanical properties diminish and the electrical conductivity does not increase proportionally, suggesting the presence of G aggregates. The biological assessment (C2C12 murine myoblasts) indicates a good biocompatibility and proliferative behavior. These results reveal a new conductive and biocompatible semi-IPN with remarkable values of electrical conductivity and ability to induce myoblast proliferation, indicating its great potential for musculoskeletal tissue engineering.

## 1. Introduction

Tissue engineering (TE) aims to engineer artificial biocompatible structures which mimic the in vivo environment of a specific tissue, allowing its regeneration and healing. This strategy combines engineered biomaterials, specific cell populations, and bioactive molecules to induce differentiation in different human tissues [1,2,3,4]. A wide variety of polymeric biomaterials can be used to generate artificial scaffolds for TE, both of natural and synthetic origin. Natural polymers are those derived from living organisms, which present excellent biocompatibility, biodegradability, and biological performance [5,6]. Nevertheless, their lack of strong mechanical properties and other tissue microstructural features might limit their applications [7]. On the other hand, synthetic polymers are artificially produced, and their properties, such as mechanical strength, chemical stability, or microstructural topography, can be modeled to match specific requirements for different tissues to enhance cell adhesion, proliferation, and differentiation [8].

Hydrogels are of special interest in TE, since they are hydrophilic crosslinked structures able to retain large amounts of water/fluids without being dissolved [9]. Several polymers have been employed for hydrogel preparation, with poly (vinyl alcohol) (PVA) being one of the most interesting. PVA is a synthetic polymer with good biocompatibility and hydrophilic behavior produced from vinyl acetate and approved for biomedical applications by the American Food and Drug Administration (FDA). Its molecular structure can be modified by crosslinking its -OH groups with different methodologies (e.g., chemical crosslinking with glutaraldehyde, physical crosslinking by freeze–thaw, etc.) to form hydrogels with excellent water-sorption capacities, widely used in different applications such as drug delivery, wound dressing, and tissue engineering [10,11,12,13]. However, its low mechanical properties and lack of cell adhesive motifs require surface modification or combination with other polymers to produce novel biomaterials with enhanced properties and broader applications [14,15]. Semi-interpenetrated polymer networks (semi-IPNs) represent an interesting approach to combine different natural and/or synthetic polymers, forming polymeric composites in which only one component is crosslinked, and the other/s remains entangled into the crosslinked matrix in a linear or branched conformation [10,16]. With a semi-IPN structure based on crosslinked PVA and a second polymer that will improve its drawbacks, the properties of the hydrogel can be maintained while enhancing mechanical properties and bioactivity (in terms of cell adhesion).

Poly(3-hydroxybutyrate-co-3-hydroxyvalerate) (PHBV) is a natural aliphatic biopolyester found in different bacteria and archaea as an internal carbon source and energy storage [17]. It is also approved by the FDA, and its excellent mechanical properties, tunable degradation, and cell adhesion capacity make PHBV one of the most studied polyhydroxyalkanoates (PHAs) in the field of TE [18,19,20,21]. Its brittleness, null antimicrobial activity, and no water sorption due to its hydrophobic nature evidence that a suitable application for PHBV would be its use in combination with hydrophilic and more flexible polymers [22]. 

In addition, conductive biomaterials have been used in TE to generate electroactive substrates able to stimulate regeneration in electrically active tissues (bone, nerve, heart, muscle) even without external electrical stimulation [23,24,25,26]. Conductive polymers, such as polyaniline (PANI), polypyrrole (PPy), poly(3,4-ethylenedioxythiophene (PEDOT), etc., and carbon nanomaterials are typically used in combination with natural or synthetic biomaterials [27,28]. Graphene (G), a characteristic 2D carbon nanomaterial, is of particular interest in the biomedical field since it presents remarkable conductivity, excellent mechanical properties, good thermal stability, and extended surface area [29,30]. Graphene itself and some of its derivates such as graphene oxide (GO) and reduced graphene oxide (rGO) have been proposed as a new approach to enhance regeneration of different electrosensitive tissues [30,31,32,33]. Hurtado et al. recently reported that the incorporation of graphene nanoplatelets into semi-IPNs of calcium alginate and PHBV significantly increased its antiviral activity against a surrogate of SARS-CoV-2, while showing a good compatibility with human keratinocyte HaCaT cells [22]. Several approaches have developed conductive PVA hydrogels for tissue engineering applications in combination with different conductive nanoparticles. For example, Wang et al. [34] described how the combination of PVA with GO and rGO increased cell attachment in comparison with pure PVA. Moreover, the mechanical and electrical properties of PVA were significantly enhanced after the addition of few-layer graphene [35]. In addition, the combination of hydrophobic polymers such as PHBV or PCL with graphene nanosheets have resulted in different nanocomposites with enhanced conductivity, mechanical properties, and good cell adhesion [36,37]. Nevertheless, the lack of hydrophilicity limits their application in tissue engineering. To our knowledge, there is no evidence in the literature about the combination of PVA/PHBV materials with G nanosheets to obtain novel nanohybrid hydrogels with electroactive properties for muscle tissue engineering.

In previous work from our research group, we developed and patented a novel semi-IPN based on PHBV/PVA with incorporated PPy nanoparticles to enhance the network’s conductivity [10,38]. The obtained nanocomposite presented a homogeneous compact structure with enhanced thermal and conductive properties, but it lacked a 3D porous structure, and the conductivity values were quite low, even with high nanoparticles content (up to 15% wt/wt). Therefore, the door was left open to further study different approaches to modify this novel hydrogel in terms of morphology, swollen properties, and electrical conductivity for its use as electroactive artificial extracellular matrix for TE. 

In the present work, we developed a new method to generate PHBV/PVA semi-IPNs with a porous 3D structure, incorporating G nanosheets to generate new 3D polymeric networks (PHBV/PVA/G) with enhanced conductivity despite using ultra-low graphene concentrations (0.2% and 0.8% wt/wt) to mimic the physiological electrical properties of musculoskeletal tissue and avoid potential cytotoxic effects. The morphological and physicochemical characterization was performed by electronic microscopy, Fourier transform infrared spectroscopy, swelling assay, thermal and mechanical analysis, and surface electrical conductivity. Finally, its biocompatibility was assessed with murine myoblasts (C2C12 cell line). 

## 2. Materials and Methods

### 2.1. Materials and Reagents

PVA (Mw 13,000–23,000 g/mol, 87–89% hydrolyzed), 1-methyl-2-pyrrolidinone (NMP) (Mw 99.13 g/mol), and G nanosheets were supplied from Sigma Aldrich-Merck, St. Louis, MO, USA. PHBV, with 2% wt of 3-hydroxyvalerate (Mw 410,000 g/mol) was purchased from Goodfellow (Huntingdon, UK). Chloroform (Mw 119.4 g/mol, 99.9% pure), glutaraldehyde (GA) (25 % wt/wt solution), methanol (Mw 32 g/mol, 99.8% pure), and sulfuric acid (Mw 98 g/mol, 95–98% pure) were supplied by Scharlab, Sentmenat, Spain. All reagents were used as received. 

### 2.2. Preparation of Semi-IPN PHBV/PVA/G Hydrogels

The materials’ synthesis was adapted from a previous work by the same research group in which a similar goal was shared: development of semi-IPN hydrogels based on PHBV/PVA, ratio 30/70 wt/wt, with conductive properties provided by filler nanoparticles. Nanoparticles of the conductive polymer PPy were added as conductive filler [10]. In this work, the aim is to obtain highly porous 3D structures with electroactive properties by using very low amounts of G nanosheets. A new method to prepare the materials was developed, based on a hybrid solvent casting–freeze-drying process.

PVA was dissolved in NMP (5% wt/wt) for 2 h at 150 °C, while PHBV was dissolved in chloroform (3% wt/wt) for 2 h at 50 °C (both with continuous stirring of 500 rpm for PHBV and 300 rpm for PVA). Then, PHBV and PVA solutions were mixed in a 30/70 PHBV/PVA ratio and the crosslinking solution (4% wt/wt GA with respect to the total PVA content) was added and stirred for 30 min at 50 °C prior to solvent casting, in order to crosslink the PVA chains, forming a hydrogel where PHBV remains entangled within the PVA matrix. This solution was composed of GA (crosslinker, 25% GA solution), methanol (quencher, diluted 50% in MilliQ water), acetic acid (pH controller, diluted 10% in MilliQ water), and sulfuric acid (catalyst, diluted 10% in MilliQ water) mixed in a 2:2:3:1 volumetric ratio [10]. Then, the solution was transferred to Petri dishes for solvent casting (24 h at 60 °C), followed by three consecutive washings with MilliQ water (three times) and three immersions in MilliQ water after 1 h, 3 h, and 24 h to remove any traces of solvent and keep the swollen state of the samples. Then, the samples were frozen at −80 °C for 24 h and subsequently lyophilized for 72 h at −80 °C under vacuum (0.1 mbar) to preserve the porous structure. 

Crosslinked PVA and pristine PHBV were used as reference. Crosslinked PVA was obtained following the same procedure but skipping the mixing step with PHBV. PHBV samples were obtained by solvent casting; the PHBV–chloroform solution was poured into a Petri dish, allowing solvent evaporation for 24 h at room temperature, followed by drying at 60 °C under vacuum to constant weight to completely remove all traces of solvent. 

A previous step was carried out to prepare the semi-IPNs of PHBV/PVA containing G nanoparticles (in the form of nanosheets). The nanoparticles (0.2% and 0.8% wt/wt relative to the total polymeric mass) were dispersed in the specific amount of NMP needed to dissolve PVA in an ultrasonic bath for 6 h, after which PVA was dissolved in the NMP–G suspension. Then, the semi-IPN PHBV/PVA/G substrates were prepared following the same protocol described for the semi-IPN without G nanosheets. 

Table 1 provides detailed information about the sample compositions of the study.

**Figure 1:**
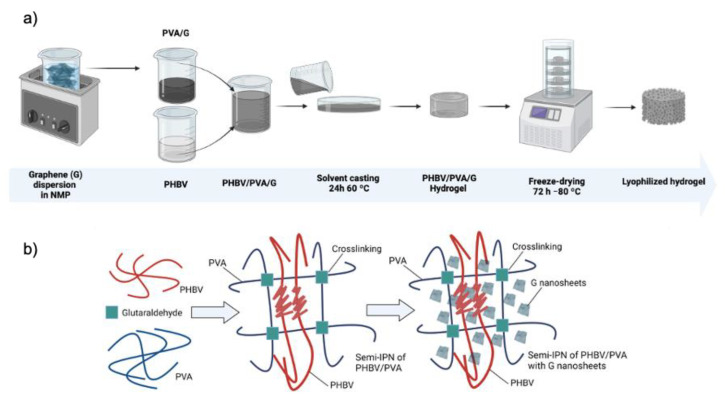
(**a**) Preparation process of the hydrogel scaffolds and (**b**) schematic diagram of the proposed PVA–PHBV–graphene hydrogel system. Created with Biorender.com.

Figure 1 shows the preparation process of the hydrogel scaffolds and the schematic diagram of the proposed PVA–PHBV–G system.

### 2.3. Morphological and Physicochemical Characterization

#### 2.3.1. Electron Microscopy

The surface and cross-section morphology of the crosslinked samples were analyzed by SEM (Zeiss ULTRA 55 Field Emission Scanning Electron Microscope (FESEM) (Carl Zeiss Microscopy, Jena, Germany)) with an accelerating voltage of 1.5–3.0 kV. The samples were coated with a platinum layer using a sputter coating (EM MED020, Leica, Wetzlar, Germany). The cross-section was observed in samples previously immersed in liquid nitrogen and cryofractured.

#### 2.3.2. Fourier Transform Infrared Spectroscopy (FTIR)

Fourier transform infrared (FTIR, Bruker ALPHA II Compact FT-IR Spectrometer, MA, USA) was used to study the surface functional groups (transmittance mode). The experiments were carried out at room temperature using 32 scans over a range of 4000–400 cm^−1^ at a resolution of 2 cm^−1^. 

#### 2.3.3. Swelling Assay

Swelling experiments were performed gravimetrically in crosslinked samples. Circular freeze-dried samples (11 mm diameter) were immersed in MilliQ water at 37 °C until equilibrium after 24 h. Redundant surface water was removed using filter paper. The experiments were performed in triplicate to ensure reproducibility. 

Samples were weighted before (*W*_0_) and after (*W*_1_) swelling, and the swelling degree (*W_eq_*) was calculated as follows:

(1) $$W_{eq}\left(\%\right)=\frac{W_{1}-W_{0}}{W_{0}}\cdot 100$$

#### 2.3.4. Differential Scanning Calorimetry (DSC)

DSC analysis was carried out in a PerkinElmer DSC 8000 (Pekin Elmer, Waltham, MA, USA) under a flowing nitrogen atmosphere (20 mL/min). After erasing the effects of any previous thermal history by heating at 220 °C for 5 min, the samples were subjected to a cooling scan down to −20 °C, followed by a heating scan from that temperature up to 220 °C, both at 20 °C/min. The glass transition temperature, *T_g_*, was calculated from the heating scan as the inflexion point of the specific heat capacity, *C_p_*, vs. temperature, which coincides with a maximum in the temperature derivative (*dc_p_*/*dT*).

The degree of crystallinity, *X_c_*(*%*), was calculated according to

(2) Xc%=∆Hmω∆Hcomp0·100

where $∆H_{m}$ is the melting enthalpy of the sample, $∆H^{0}$ is the melting enthalpy for the 100% crystalline component of the semi-IPN, and ω is the component weight fraction.

#### 2.3.5. Thermogravimetric Analysis (TGA)

TGA was used to study the thermal decomposition kinetics. Vacuum-dried samples (5–10 mg weight) were heated from 30 to 600 °C at a rate of 10 °C/min using a Mettler Toledo TGA 2 (SF) system (Mettler Toledo, Columbus, OH, USA). The mass of the samples was constantly measured as a function of temperature.

#### 2.3.6. Mechanical Properties

Dynamic mechanical analysis was performed on a DMA 8000 (PerkinElmer, Waltham, MA, USA) at a frequency of 1 Hz on circular samples (11 mm diameter) in an immersion bath (MilliQ water) in compression mode. The storage modulus (*E*′) and loss modulus (*E*″) were measured in the temperature range 36 to 38 °C at a heating rate of 1 °C/min. 

#### 2.3.7. Electrical Conductivity

To study the conductivity of the developed hydrogels, a flat structure was needed to ensure good contact with the probes. Thus, the hydrogels were first hydrated, subsequently placed between glass covers, and dried in a vacuum until constant weight to remove any water residue. 

The electrical surface resistance (*R_S_*) of flat circular samples (10 mm diameter) was measured on a T2001A3-EU four-point probe system-EU plug (Ossila Limited, Sheffield, UK). The electrical conductivity (*σ*) was calculated according to the following expression:

(3) $$\frac{1}{R_{S}\times l}$$

where *l* is the film thickness, measured with a digital caliper (Acha, Spain). The measurements were performed in triplicate to ensure reproducibility. 

### 2.4. Cytotoxicity Assessment

The cytotoxicity of the hydrogels was tested by performing a direct MTS assay. This colorimetric test is based on the reduction of the tetrazolium salt (3-[4,5, dimethylthiazol-2-yl]-5-[3-carboxymethoxy-phenyl]-2-[4-sulfophenyl]-2H-tetrazolium) (MTS) by the mitochondrion NADPH-dependent dehydrogenases, only active in viable cells. This metabolic process results in the generation of a soluble formazan dye whose absorbance can be quantified, providing information on the viability of cells cultured on the material. 

Murine myoblasts (C2C12 cell line) were cultured on plasma-treated 24-well culture plates at 20.000 cells/cm^2^ with growth media (DMEM high glucose, 10% fetal bovine serum (FBS) and 1% penicillin/streptomycin antibiotic mix (P/S)) for 48 h at 37 °C and 5% CO_2_ to allow cell confluence. Cell substrates (c-PHBV/PVA, c-PHBV/PVA 0.2% G, and c-PHBV/PVA 0.8% G) were first sterilized with 3 consecutive washings (5 min) with 70% ethanol and one last washing (10 min) with MilliQ water and then placed above cells in direct contact (3 biological replicates with 3 technical replicates for each biological replicate, material, and time). These conditions were kept for 24, 48, and 72 h. At every time point, the materials were carefully removed, and the culture medium was replaced by DMEM without phenol red with an MTS 1:10 dilution and left incubating for 2 h to allow MTS metabolization by cultured cells. After that, the resulting formazan-dyed media absorbance was measured in a microplate reader (Victor Multilabel Plate Reader, Perkin Elmer, MA, USA) at 490 cm^−1^. Cell viability was calculated as follows:

(4) $$Cellviability\left(\%\right)=\frac{ODtest}{ODcontrol}*100$$

where OD corresponds to the optical density of the tested material and OD control is the optical density of the negative control. The negative control (cell life) consisted of C2C12 seeded in growth media without any material. C2C12 cells seeded in growth media, with 2% triton X-100 solution inoculation 1 h before MTS inoculation (at every time point), were used as positive control (cell death). 

### 2.5. Statistical Analysis

Statistical analysis was carried out by one-way ANOVA tests on all samples using GraphPad Prism 8.0 software, with three replicates per condition unless noted. Data are presented as mean ± standard deviation. If significant differences were noted between samples, Tukey tests were used to perform pairwise comparations with a 95% confidence level (*p* < 0.05). 

## 3. Results and Discussion

### 3.1. Microstructure and FTIR Analysis of the Nanohybrid Hydrogels

**Figure 2:**
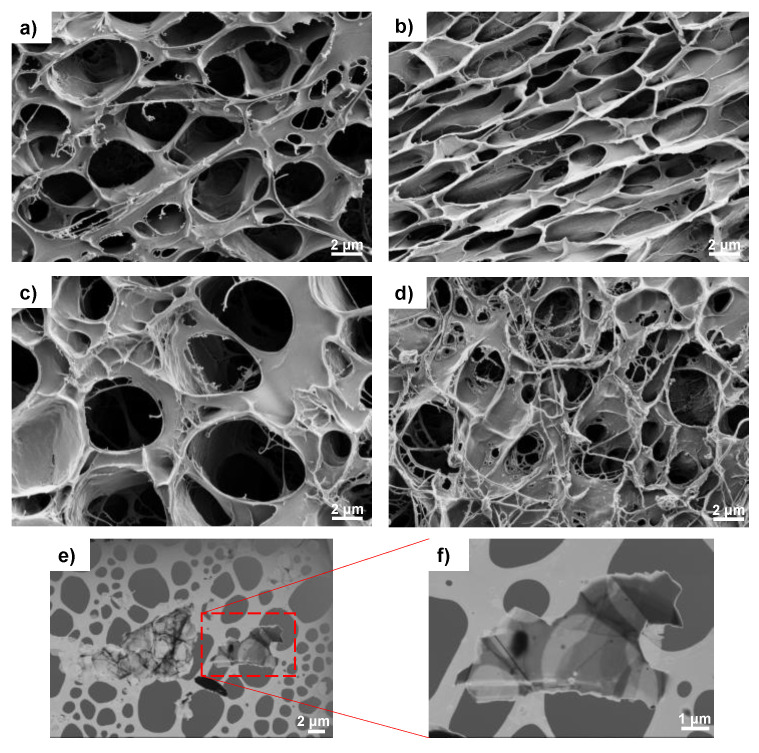
(**a**) High-resolution FESEM surface images of the hydrogels: (**a**) c-PVA, (**b**) c-PHBV/PVA, (**c**) c-PHBV/PVA 0.2% G, and (**d**) c-PHBV/PVA 0.8% G. (**e**,**f**) Aggregated and detailed single-form HRFESEM pictures of graphene nanosheets on a TEM grid previously dispersed in THF. To preserve the porous structure, the samples were immersed in MilliQ water for 24 h, frozen in liquid nitrogen, and stored at −80 °C until freeze-drying for 24 h. Magnification (**a**–**d**): 5 kX, (**e**): 3.5 kX, (**f**): 10.8 kX.

**Figure 3:**
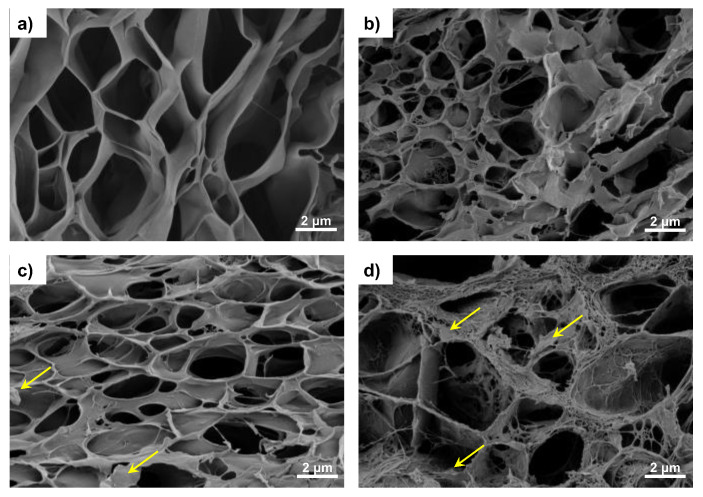
High-resolution FESEM cross-section pictures of the hydrogels: (**a**) c-PVA, (**b**) c-PHBV/PVA, (**c**) c-PHBV/PVA 0.2% G, and (**d**) c-PHBV/PVA 0.8% G. Cross-sections were obtained after cryogenic cut with liquid nitrogen. Magnification: 7 kX.

The morphology of the samples is depicted in Figure 2 (surface) and Figure 3 (cross-section). The combined technique (solvent casting–freeze-drying) produced a highly interconnected porous structure with pores between 0.5 and 10 µm (crosslinked PVA (Figure 2a and Figure 3a) is included as reference). After the addition of 30% of the hydrophobic polymer PHBV, the porous structure of the swollen samples is maintained, although a decrease in pore size and the presence of small threads (more noticeable in the surface images can be observed (Figure 2b and Figure 3b). The reduction in the pore size indicates a decrease in the swollen capacity, which is analyzed below. However, the semi-IPN shows a homogeneous structure, and the small threads can be produced by traces of PHBV or PVA not integrated into the structure of the semi-IPN during the solvent evaporation. 

The addition of G nanosheets does not alter the porous structure, and the mixture of the solvents water and chloroform, which are miscible, favors the dispersion of G nanosheets, as observed in Figure 3c,d (see the yellow arrows in the cross-section images). The methodology used to prepare the samples results in highly porous structures, both in the surface (Figure 2c,d) and the cross-section (Figure 3c,d). It has been reported that nano/micropores promote cell adhesion by increasing the surface area and facilitating nutrient diffusion. In addition, microporous structures (a few microns) play an important role at the cellular level, improving the biological performance of artificial cell substrates [39].

**Figure 4:**
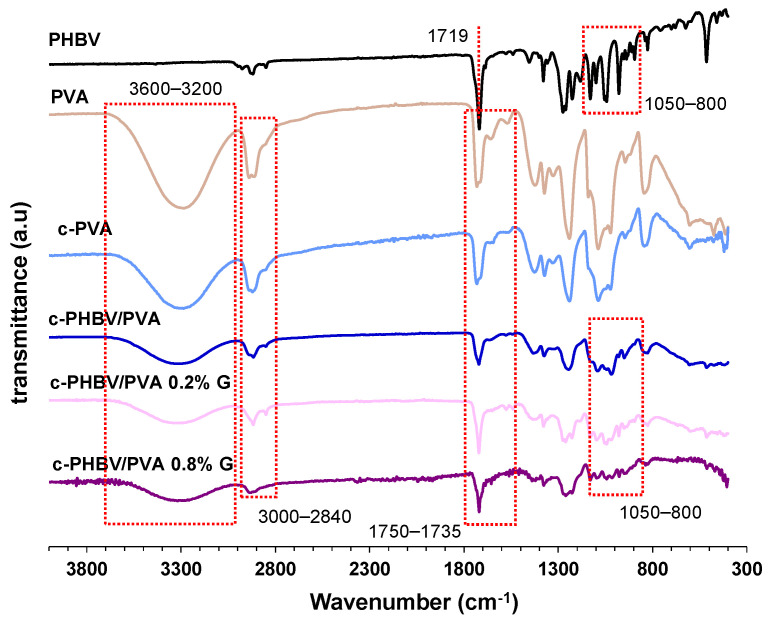
Fourier transform infrared spectroscopy (FTIR) spectra in the region 4000–300 cm^−1^ of the semi-IPN with and without G nanoparticles. Neat PVA, crosslinked PVA (c-PVA), and neat PHBV are also included as reference samples.

FTIR spectra of the semi-IPN PHBV/PVA hydrogel with and without G nanosheets are shown in Figure 4. Pristine PVA, crosslinked PVA (c-PVA), and PHBV are included as reference. The spectra of neat PVA and c-PVA show the major vibrational peaks: the stretching of OH groups (band between 3600 and 3200 cm^−1^) related to the intermolecular and intramolecular hydrogel bonds, the vibrational band related to the stretching of the C–H bond in the alkyl group (2840–3000 cm^−1^), and the stretching of the C=O and C–O bonds (1750–1735 cm^−1^). PVA crosslinking with bifunctional crosslinker GA produces the formation of acetal bridges between the hydroxyl groups in PVA and the aldehyde groups of GA [40,41]. The reduction in the intensity of the OH band (3600–3200 cm^−1^) in the c-PVA sample evidences the crosslinking by the formation of acetal. The PHBV spectrum shows the characteristic -C–O–C- stretching vibration (800 and 1050 cm^−1^) and a band related to the C=O stretching at 1719 cm^−1^ [42]. The spectrum of the semi-IPN PHBV/PVA reveals the peaks related to both PVA and PHBV. The reduction of the intensity of the OH band (3600–3200 cm^−1^) is indicative of the reduced number of the hydroxyl groups after the addition of the hydrophobic PHBV [10]. As graphene does not have functional groups [43], the FTIR spectra of the hydrogels with G nanosheets do not show differences to that of the semi-IPN without nanoparticles, confirming that there is no interaction between the graphene nanosheets and the polymer matrix. 

### 3.2. Physical Characterization

#### 3.2.1. Swelling Properties

**Figure 5:**
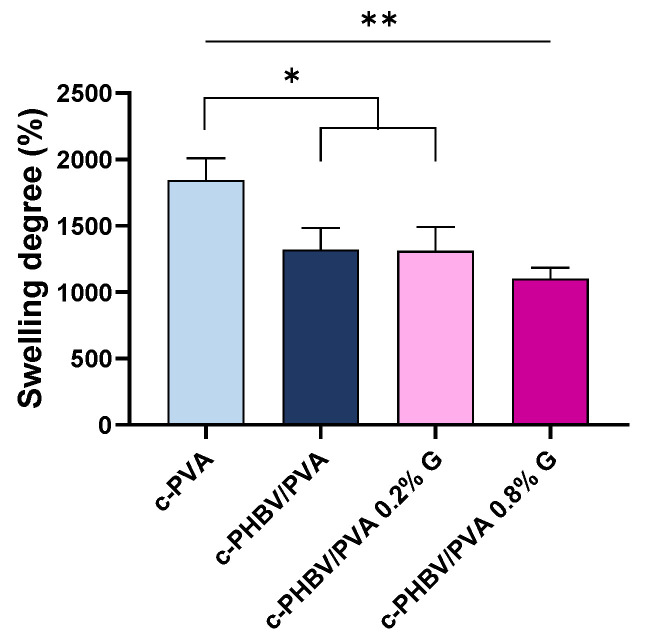
Equilibrium swelling degree of the engineered hydrogels after 24 h immersed in MilliQ water. Graph shows mean ± standard deviation. (*) and (**) indicate significant differences (*p* < 0.05 and *p* < 0.01, respectively). Crosslinked PVA (c-PVA) is included as reference.

The equilibrium swelling degree of the semi-IPN PHBV/PVA with and without G nanosheets is included in Figure 5. As expected, the swelling capacity of the semi-IPN c-PHBV/PVA decreased significantly (*p* < 0.05) due to the presence of 30% of the hydrophobic PHBV compared to crosslinked PVA (reference), although due to its high porous structure, the swelling degree is still higher than 1200%. The addition of 0.2% and 0.8% of G nanosheets does not have a significant impact on the swelling capacity compared with the semi-IPN without G nanoparticles, although statistically significant differences remain with respect to crosslinked PVA (*p* < 0.05 and *p* < 0.01 for the nanocomposites with 0.2% and 0.8% of G nanosheets, respectively). This behavior indicates that low percentages of G nanosheets (<1%) distributed within the polymer matrix, despite being highly hydrophobic, are not enough to affect the swelling capacity of the nanocomposites. The highly porous structure is maintained after the addition of G nanosheets, allowing swelling degrees higher than 1100%. 

#### 3.2.2. Thermal Behavior and Thermal Degradation

**Figure 6:**
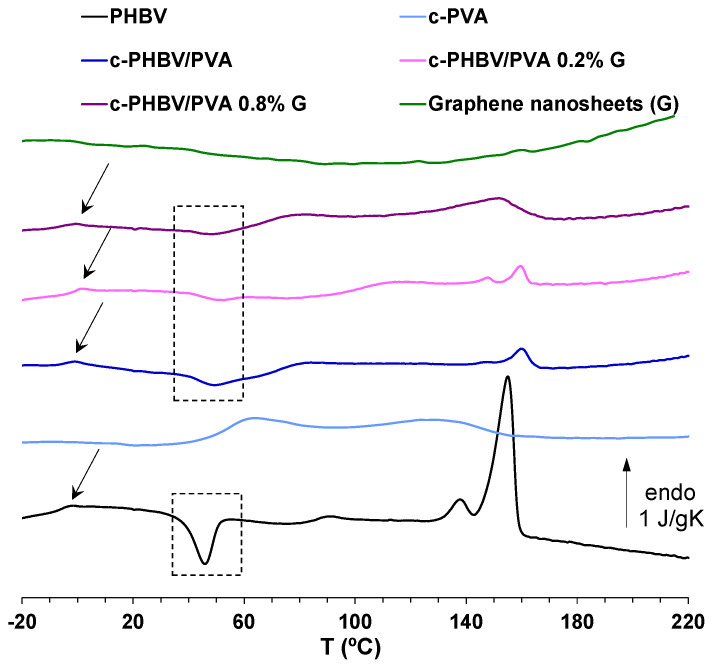
DSC thermograms at a rate of 20 °C/min of c-PHBV/PVA and c-PHBV/PVA with graphene nanosheets (0.2% and 0.8%). Normalized heat flow on heating. Neat PHBV, crosslinked PVA (c-PVA), and G nanosheets are included as reference.

Figure 6 shows the DSC scan (on heating) of the material system, and the characteristic thermal parameters obtained from the thermogram (glass transition temperature (*T_g_*), cold crystallization temperature (*T_c_*_(*cold*)_), melting temperature (*T_m_*), melting enthalpy (Δ*H_m_*), degree of crystallinity (*X_c_*(%)) are collected in Table 2. Crosslinked PVA and neat PHBV (flat sample) are included as references. Neat PHBV shows the glass transition process around 0 °C (*T_g_*), an exotherm peak related to cold crystallization (*T_c_*_(*cold*)_ ≈ 45 °C) followed by a multiple peak associated with the melting process (*T_m_* ≈ 155 °C). As expected, and consistent with previous results, no melting process was observed in crosslinked PVA, pointing out that crystallization is prevented by the crosslinking to which the sample was subjected. The glass transition process of crosslinked PVA can be observed in the interval between 45 and 65 °C [10]. The semi-IPNs with and without G nanosheets show different processes identified in both pristine PHBV and crosslinked PVA. The glass transition of the PHBV chains can be observed in the composites (see the arrows in Figure 4). In addition, the thermogram shows a cold crystallization process in the same interval as the crosslinked PVA glass transition, followed by a small melting process. The cold crystallization, between 30 and 50 °C, and the melting, from 130 to 170 °C, can be observed in the same intervals as the PHBV cold crystallization and melting, respectively, suggesting that PHBV chains are involved in these processes. These results indicate that the combined methodology used to prepare the hybrid hydrogels induces phase separation between the hydrophilic PVA and the hydrophobic PHBV.

However, the semi-IPN morphology (at micrometric scale) is homogeneous, as shown in the microscopy images (Figure 1 and Figure 2), which indicate that the semi-IPN presents microphase separation, with microdomains of PHBV highly intertwined with the PVA network. As mentioned above, in a previous study, where PHBV/PVA semi-IPN with PPy nanoparticles were prepared using a different methodology (solvent casting in two phases at different temperatures), the thermal analysis (DSC results) showed a single glass transition process located between those of the neat components, indicating a good miscibility of the blend, with no phase separation [10]. Using this new method to obtain hydrogels with higher porosity, the microphase separation might have occurred during the washing and swelling in water applied between the solvent evaporation and the freeze-drying, causing PHBV chains (of hydrophobic character) to clump together in the presence of water. The PHBV chains inside the microdomains, despite the restriction imposed by the PVA network, can reorganize and crystallize, albeit to a lesser extent than pristine PHBV, as shown in Figure 6. The degree of crystallinity of the PHBV, *X_c_*(*%*), was calculated according to equation 2. The enthalpy $∆H_{m}$ was obtained from the area of the melting peak, taking a baseline from 125 to 170 °C (Figure 4). Considering the low percentage of hydroxyvalerate in the copolymer PHBV (2%), it can be assumed that the crystallization process is related to poly-hydroxybutyrate (PHB); therefore, $\Delta H_{comp}^{0}=∆H_{PHBV}^{0}\approx ∆H_{PHB}^{0}=132\;J/g$. 

The degree of crystallinity of PHBV microdomains in the semi-IPN decreases significantly compared to pristine PHBV (from 16.1 to 2.6%). The procedure used in this study, despite the microphase separation, produces highly porous hydrogels with a good stability in aqueous environments. The thermal behavior of the semi-IPN after the addition of G nanosheets is not significantly changed. The cold crystallization process and melting of the PHBV microdomains can be observed, although the crystallinity of these microdomains increased from 2.6% for the semi-IPN PHBV/PVA without G nanosheets to 4.7% and 12.3% after the addition of 0.2% and 0.8% of G nanoparticles, respectively. These results indicate that the G nanosheets, distributed within the semi-IPN, act as nucleating agent for PHBV. The increase of G nanosheets from 0.2 to 0.8% (four times higher), raises the crystallinity of the PHBV microdomains, although to a lesser extent (2.6-fold), suggesting the presence of aggregates when the quantity of nanoparticles increases. The structure of the PVA–PHBV–G hydrogel system is shown in Figure 1b. 

**Figure 7:**
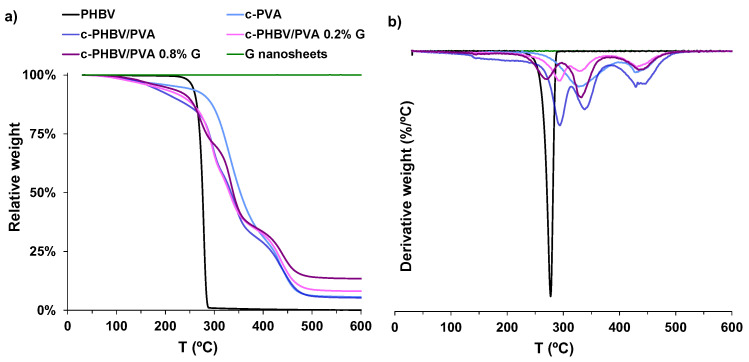
(**a**) Thermogravimetric analysis of the semi-IPN with and without G nanosheets. (**b**) Derivative of the weight loss. Neat PHBV and crosslinked PVA (c-PVA) are included as reference.

The thermal degradation, analyzed by thermogravimetry for temperatures up to 600 °C, is shown in Figure 7. PHBV presents a one-stage degradation at temperatures above 250 °C, related to the hydrolysis and chain scission that produce crotonic acid [44]. Crosslinked PVA shows a three-stage profile, related to moisture vaporization (50–130 °C), followed by two processes at higher temperatures related to the dehydration of hydroxyl groups and hydrocarbon products degradation (above 250 °C) and, finally, the breakage of the main chain (above 400 °C) [45,46]. As expected, the degradation profile of the semi-IPN PHBV/PVA is located between those of the components, closer to crosslinked PVA (the major component of the semi-IPN) and with a similar behavior. The decomposition temperature at which weight loss was 50% (*T_d−_*_50%_), included in Table 2, increases from 276.3 °C for pristine PHBV to 334.5 °C for the semi-IPN, close to 352.8 °C obtained for the crosslinked PVA sample. The addition of G nanosheets does not significantly change the degradation profile, which is similar to that of the semi-IPN without G nanosheets, with *T_d−_*_50%_ in the same temperature range (331.1–339 °C). However, at temperatures above 450 °C, a residual weight that increases with G nanosheets content can be observed, which suggests that G nanosheets contribute to improve thermal stability at high temperature, as previously reported [47]. 

#### 3.2.3. Mechanical and Electrical Properties

**Figure 8:**
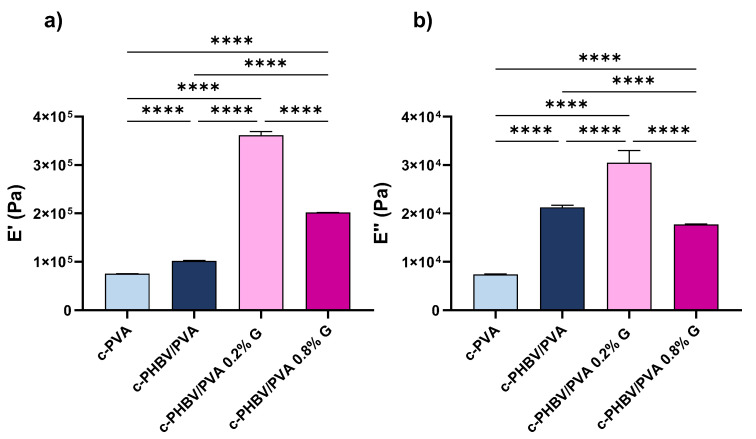
Dynamical mechanical analysis (compression mode). (**a**) Storage modulus (*E*′) and (**b**) loss modulus (*E*″) at 37 °C for c-PHBV/PVA and nanohybrid hydrogels with 0.2% and 0.8% of G nanosheets. Crosslinked PVA (c-PVA) is included as reference. Graph shows mean ± standard deviation; (****) indicates significant differences (*p* < 0.001).

The measurement of mechanical properties by dynamical mechanical analysis is a first approach to evaluate the applications of engineered scaffolds [48,49,50]. The complex modulus, storage modulus (*E*′), and loss modulus (*E*″), obtained in a wet environment (immersion bath) at 37 °C to simulate physiological conditions, are shown in Figure 8. Statistically significant differences were found between all the samples (*p* < 0.001). As expected, both *E*′ and *E*″ increased from 7.52 × 10^4^ Pa and 7.26 × 10^3^ Pa, respectively, for crosslinked PVA (considered as reference) to 1.02 × 10^5^ Pa and 2.08 × 10^4^ Pa for the semi-IPN, due to the presence of 30% of PHBV, that possesses higher mechanical strength. This enhancement indicates that the PHBV chains, located between the PVA network, reinforce the structure of the composite, despite the microphase separation between both components (as stated by the thermal characterization). The addition of small percentages of G nanosheets significantly increases the mechanical properties, as reported elsewhere [51,52]. In this study, the increase of the storage modulus after the addition of only 0.2% of G nanosheets increased almost fourfold (from 1.02 × 10^5^ Pa for the semi-IPN to 3.87 × 10^5^ Pa for the nanocomposite), due to the interfacial interactions between the nanoparticles and the polymer matrix, which also suggest a good dispersion within the matrix. However, after incorporating 0.8% of G nanosheets, the enhancement in the storage module decreases until 2.02 × 10^5^, although it is still above of that of the semi-IPN without nanofiller. The loss modulus of the c-PHBV/PVA 0.8 G also decreases, with a value slightly lower than that of the semi-IPN without nanoparticles. These results suggest that, at this concentration, G nanosheets, due to strong van der Waals forces, tend to agglomerate, which results in a weaker interaction with the matrix and a less effective stress transfer [51,53]. Yang et al. prepared hybrid membranes based on polydimethyl siloxane–G nanosheets and reported that when the nanofiller content reached 0.5% wt, the agglomeration of the nanoparticles reduced the interfacial area, hindering the stress transfer across the G nanosheets–polymer matrix interface [54], in good agreement with the obtained results.

**Figure 9:**
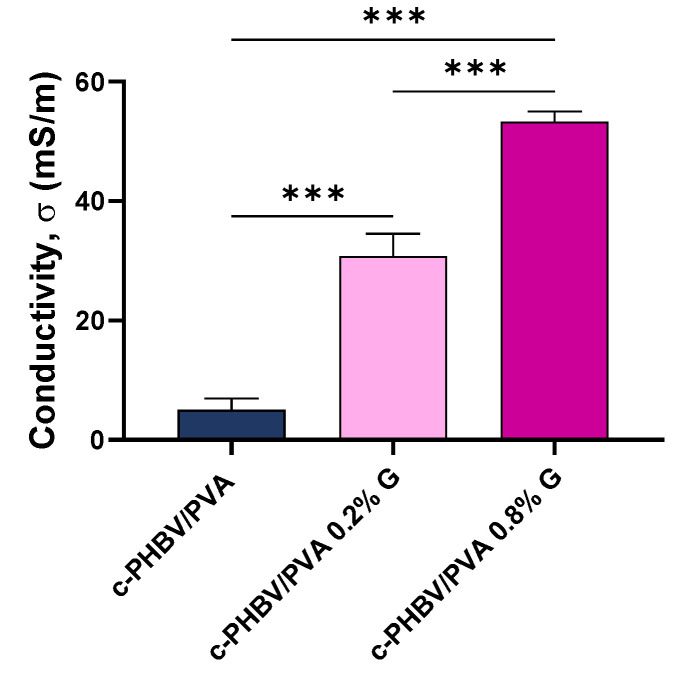
Electrical surface conductivity of the semi-IPNs with and without graphene nanosheets. Graph shows mean ± standard deviation; (***) indicates significant differences (*p* < 0.001).

The electrical surface conductivity, obtained by equation (3) and depicted in Figure 9, shows a statistically significant increase (*p* < 0.001) after the addition of G nanosheets compared to the semi-IPN without nanoparticles as expected. Significant differences were also found between nanocomposites with 0.2% and 0.8% of G nanosheets (*p* < 0.001). The conductivity increases more than 600% after the addition of 0.2% of nanofiller (from 5.0 to 30.7 mS/m). When 0.8% of G nanosheets are added, the surface conductivity increases to 53.2 mS/m; however, increasing the nanofiller content four times results in a 1.7-fold enhancement of surface conductivity (compared to 0.2% G nanosheets). Thus, the noncorrelation between the nanofiller content and conductivity may be related to the presence of nanoparticles aggregates, which is consistent with the previously analyzed thermal behavior and mechanical performance. 

A particular challenge in musculoskeletal tissue engineering is to engineer constructs that mimic the properties of the native tissue. Mechanical and electrical signaling cues (skeletal muscle is considered an electrosensitive tissue) are considered as important factors for successful muscle regeneration, together with topography and porosity [55,56]. The mechanical properties of the semi-IPN with and without G nanosheets are in the range of those reported for the elastic modulus of skeletal muscle tissue (30–8000 kPa) [57]. Regarding electrical properties, the values of surface conductivity (between 5.03 to 53.25 mS/m), which may be effective in the transference of electrical cues, are also in the same range as the skeletal muscle tissue (σ ≈ 10^−3^ S/m) [58] and other engineered conductive cells substrates prepared for musculoskeletal regeneration that have shown an improved cell response [59,60]. 

### 3.3. Biocompatibility

**Figure 10:**
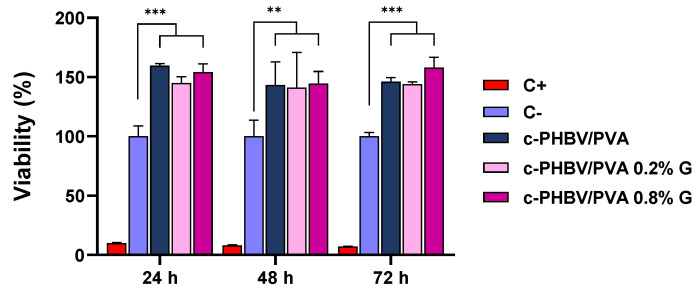
Cell viability after 24, 48, and 72 days from MTS assay. Semi-IPN c-PHBV/PVA and nanohydrogels with 0.2% and 0.8% of graphene nanosheets. Positive control: growth medium with latex extract as cytotoxic agent. Negative control: growth medium. Graph shows mean ± standard deviation; (**) and (***) indicate significant differences (*p* < 0.01 and *p* < 0.001, respectively).

The biocompatibility of the biomaterial was assessed for C2C12 murine myoblast exposed to direct contact with the materials (semi-IPN PHBV/PVA without G nanosheets and with 0.2% and 0.8% of nanoparticles). Both pristine components, PHBV and PVA, are biocompatible polymers, widely used for biomedical applications [14,61,62,63]. On the other hand, it has been reported that carbon nanomaterials may induce cytotoxic effects in a dose-dependent manner [64], although the shape (layer or nanotube, for example) and the processing techniques to prepare the biomaterials play a decisive role [65,66]. The cell viability after 24, 48, and 72 h are shown in Figure 10. 

All the materials show viability values over the negative control (cells seeded with growth medium and without any materials, considered as 100% viability) in the evaluated times. Statistically significant differences were found for all the evaluated times (*p* < 0.001 for 24 and 72 h, and *p* < 0.01 for 48 h) between the cell substrates (with and without nanoparticles). The semi-IPN shows values higher than 140% for the three times assessed, with significant differences compared to the negative control, indicating cell proliferative activity, even after only 24 h of culture. The addition of 0.2% and 0.8% of G nanosheets maintains high values of cell viability, in agreement with previous results, where small amounts of G nanosheets entangled in polymeric matrices were not cytotoxic [37,67] and promoted proliferation [68]. The high values of viability in the evaluated times imply that the nanohybrid hydrogels do not lose the proliferative capacity. 

## 4. Conclusions

The engineered conductive hydrogels developed in this study are safe in terms of cytotoxicity and possess high bioaffinity with C2C12 cells, thus promoting cell proliferation. These porous hydrogels, developed by a hybrid solvent casting–freeze-drying method, and 0.2% of G nanosheets possess interconnected porous structure, high water-absorption capacity, thermal stability, and suitable mechanical and conductive properties (in the range of skeletal muscle tissue). Although further physicochemical and biological studies are needed to explore the applications of these novel electroactive semi-IPN PHBV/PVA/G hydrogels, the results of this study postulate them as promising candidates for electroactive tissue regeneration applications, particularly muscle tissue engineering.

## Figures and Tables

**Table 1:** Notation of the samples.

Identification	Description
PHBV	PHBV 3% wt/wt film (solvent casting)
c-PVA	PVA 5% wt/wt film crosslinked with 4% wt/wt GA (solvent casting–freeze-drying)
c-PHBV/PVA	30% PHBV/70% PVA semi-IPN crosslinked with 4% wt/wt GA (solvent casting–freeze-drying)
c-PHBV/PVA 0.2% G	30% PHBV/70% PVA semi-IPN crosslinked with 4% wt/wt GA + 0.2% G nanosheets (solvent casting–freeze-drying)
c-PHBV/PVA 0.8% G	30% PHBV/70% PVA semi-IPN crosslinked with 4% wt/wt GA + 0.8% G nanosheets (solvent casting–freeze-drying)

**Table 2:** Glass transition temperature (*T_g_*), cold crystallization temperature (*T_c_*_(*cold*)_), melting temperature (*T_m_*), melting enthalpy (Δ*H_m_*), degree of crystallinity (*X_c_*(%)), and decomposition temperature at which weight loss was 50% (*T_d−_*_50%_).

Sample	*T_g_* (°C)	*T_c_*_(*cold*)_ (°C)	*T_m_* (°C)	Δ*H_m_* (J/g)	*X_c_* (%)	*T_d−_*_50%_ (°C)
PHBV	−3.6	46	156	21.2	16.1	276.3
c-PVA	57	-		-	-	352.8
c-PHBV/PVA	−3.5	50	162	1.0	2.6	334.5
c-PHBV/PVA 0.2% G	−2	50,5	160	1.8	4.7	331.1
c-PHBV/PVA 0.8% G	−3.1	48	156	4.8	12.3	339.2

## Data Availability

Data will be made available on request.
